# Impact of an acute heat shock during *in vitro* maturation on interleukin 6 and its associated receptor component transcripts in bovine cumulus-oocyte complexes

**DOI:** 10.1590/1984-3143-ar2020-0221

**Published:** 2021-02-16

**Authors:** Julia R. Rowinski, Louisa A. Rispoli, Rebecca R. Payton, Liesel G. Schneider, F. Neal Schrick, Kyle J. McLean, J. Lannett Edwards

**Affiliations:** 1 Department of Animal Science, The University of Tennessee, Institute of Agriculture, AgResearch, Knoxville, TN, USA; 2 Cincinnati Zoo & Botanical Garden, Cincinnati, OH, USA

**Keywords:** cumulus-oocyte complex, heat shock, Interleukin 6, oocyte maturation, progesterone

## Abstract

An acute heat stress event after the LH surge increased interleukin 6 (IL6) levels in the follicular fluid of the ovulatory follicle in hyperthermic cows. To examine direct consequences of a physiologically-relevant elevated temperature (41.0°C) on the cumulus-oocyte complex (COC), IL6 transcript abundance and related receptor components were evaluated throughout *in vitro* maturation. Heat-induced increases in IL6 were first noted at 4 hours of *in vitro* maturation (hIVM); peak levels occurred at 4.67 versus 6.44 hIVM for 41.0 and 38.5°C COCs, respectively (SEM = 0.23; P < 0.001). Peak IL6ST levels occurred at 6.95 versus 8.29 hIVM for 41.0 and 38.5°C, respectively (SEM = 0.23; P < 0.01). Transcript for LIF differed over time (P < 0.0001) but was not affected by 41.0°C exposure. Blastocyst development after performing IVF was not affected by 41.0°C exposure for 4 or 6 h. When limiting analysis to when IL6 was temporally produced, progesterone levels were only impacted by time and temperature (no interaction). Heat-induced shift in the temporal production of IL6 and IL6ST along with its impact on progesterone likely cooperate in heat-induced hastening of meiotic progression described by others.

## Introduction

Dairy cows lose the ability to maintain body temperature when temperature-humidity index approaches 72 (Armstrong, 1994). In moderate to severe instances of environmental heat stress, cow body temperature may reach or exceed 41.0°C (Gaalaas, 1945; Seath and Miller, 1946; Roman-Ponce et al., 1977; Turner, 1982; Elvinger et al., 1991; Ealy et al., 1993). This level of hyperthermia is problematic because for each 1°C increase in rectal temperature pregnancy rate decreases by ~25% (Ulberg and Burfening, 1967). Hyperthermia occurring at or near the time of breeding during chronic periods of heat stress is especially problematic. Cows having elevated rectal temperatures before artificial insemination are more likely to return to service and have lower conception rates (Fallon, 1962). Hyperthermia related decreases in fertility are not limited to Holstein cows but are problematic in other breeds (Dunlap and Vincent, 1971), and even *Bos indicus* cattle (Zakari et al., 1981).

Mechanisms underlying heat-induced reductions in fertility are multifactorial, and to some extent relate to direct effects of elevated body temperature on maternal environment (e.g., ovulatory follicle components) and the cumulus-oocyte complex (COC) resident within (Edwards and Hansen, 1996; Lawrence et al., 2004; Zhandi et al., 2009). Related to impacting ovulatory follicle components, Rispoli et al. (2019) examined the follicular fluid proteome of lactating dairy cows that became hyperthermic as a result of an acute heat stress event occurring after a pharmacologically-induced LH surge. Hyperthermic cows had increased levels of IL6 in the follicular fluid of the ovulatory follicle (Rispoli et al., 2019). Because circulating levels were similar in cows maintained in thermoneutral and heat stress conditions (Rispoli et al., 2019), we hypothesized that heat-induced increases in follicular fluid levels of IL6 likely originated from ovulatory follicle components. In support of this notion, IL6 is produced by the mural granulosa cells (murine: (Liu et al., 2009), porcine: (Faundez et al., 2015)) and the cumulus-oocyte complex (human: (Zolti et al., 1991; Machelon et al., 1994), murine: (Liu et al., 2009), ovine: (Zhao et al., 2012), bovine: (Tscherner et al., 2018)).

Towards functional significance, Liu et al. (2009) showed that the addition of IL6 to murine COCs during *in vitro* maturation improved success of embryo transfers by increasing number of pups born. Other efforts using ovine (Zhao et al., 2012), bovine (Faundez et al., 2014) and porcine (Faundez et al., 2015) COCs noted improvements in meiotic progression (i.e., metaphase I or metaphase II) with the IL6 addition to the maturation medium. Effects of IL6 appear dose dependent when added during *in vitro* maturation. Zhao et al. (2012) demonstrated that a lower dose of IL6 (10 ng/mL) increased maturation rates of ovine COCs, whereas a higher dose (100 ng/mL) reduced maturation rates and impaired subsequent embryo development.

Mindful of effects to promote meiotic maturation (murine: (Liu et al., 2009), bovine: (Faundez et al., 2014), porcine: (Faundez et al., 2015)) and cumulus expansion (murine: (Liu et al., 2009; Wang et al., 2014), human: (Clark et al., 2011), porcine: (Faundez et al., 2015)), initial efforts of study one focused on examining *IL6* abundance in the cumulus-oocyte complex throughout *in vitro* maturation when directly exposed to the physiologically-relevant elevated temperature of 41.0°C. Because receptor mediated signaling is dependent on forming a complex with the IL6 signal transducer (Hibi et al., 1990; Mackiewicz et al., 1992; Heinrich et al., 2003; Wolf et al., 2014), the relative abundance of the IL6 receptor and its associated signal transducer (IL6ST) was also examined in COCs throughout *in vitro* maturation. Noting that a major consequence of 41.0°C exposure at the beginning of maturation was to shift the temporal production of *IL6ST*, additional effort was put forth to examine the relative abundance of another member of the IL6 family of cytokines (i.e., leukemia inhibitory factor; LIF). After receptor binding, IL6ST is also utilized for LIF based-signal transduction (Gearing et al., 1991; Tscherner et al., 2018). Like IL6, LIF has been shown by others to affect oocyte maturation (Dang‐Nguyen et al., 2014; Mo et al., 2014; Wang et al., 2019).

## Materials and methods

### Collection and *in vitro* maturation of bovine cumulus-oocyte complexes

Reagents and chemicals were obtained from MilliporeSigma (St. Louis, MO, USA) unless indicated otherwise. Oocytes were collected from abattoir-derived ovaries (Lawrence et al., 2004) located in Gaffney, South Carolina, USA (Brown Packing Co., Inc). Media were prepared per Rispoli et al. (2011). Folltropin-V (FSH) was obtained from Vetrepharm Canada, INC. (London, ON, Canada); same batch was used throughout. Cumulus-oocyte complexes with compact cumulus cell vestments and homogenous ooplasm underwent *in vitro* maturation (Study 1: ~30 COCs per 0.5 ml maturation medium in polystyrene tubes; Sarstedt AG and Co., Nümbrecht, Germany; Study 2: 29 to 45 COCs (mean = 34.3 ± 0.66) per 0.5 mL in 4-well Nunc culture dishes; Thermo Fisher Scientific, Waltham, MA, USA). Incubator temperatures were verified before and during different studies using mercury thermometers sealed in media-filled bottles.

### Study one: Interleukin 6, IL6 receptor, signal transducer and LIF transcripts during *in vitro* maturation in COCs matured at 38.5 or 41.0°C

**Figure 1:**
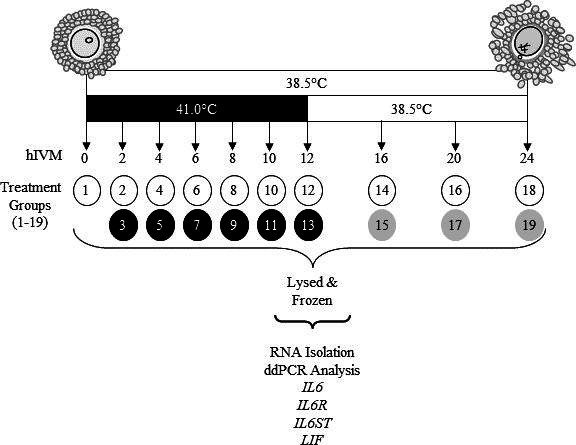
Schematic of study design. At 2, 4, 6, 8, 10, 12, 16, 20, or 24 hIVM subsets of COCs matured at 38.5°C (designated by white circles) or 41.0°C (first 12 designated by black circles, then moved to 38.5°C designated by gray circles) were removed from culture, washed, lysed before storage at -80°C until RNA extraction and subsequent RNA analyses.

Cumulus-oocyte complexes were matured at 38.5 or 41.0°C (exposure to 41.0°C was restricted to first 12 h only; thereafter COCs were transferred to 38.5°C). At 2, 4, 6, 8, 10, 12, 16, 20 and 24 h *in vitro* maturation (hIVM) subsets of COCs were removed from culture and kept separate by treatment (Figure 1; 2 × 9 factorial treatment arrangement). A subset of COCs was also processed soon after removal from ovary to provide a 0 hIVM group. Per each time period, COCs were washed twice in Dulbecco’s phosphate buffered saline containing 0.1% polyvinyl alcohol and pelleted (600 x g, 5 min). After supernatant removal, COCs were lysed in extraction buffer (Quick-RNA Kit; Zymo Research, Irvine, CA, USA) and stored at -80°C until RNA isolation. Maturation medium that was conditioned by COCs during culture was centrifuged (5 min, 3000 x g); supernatant was stored at -20°C. Cumulus-oocyte complexes were collected from ovaries on four different days with 3,840 total COCs being utilized. On a given day’s collection, two different pools of 30 COCs matured at 38.5 and 41°C were evaluated at 0, 4, 8, 12, 16, 20, and 24 hIVM resulting in a total of 8 observations per these treatment combinations. Related to 2, 6, and 10 hIVM, only one group of 30 COCs were matured at 38.5 and 41°C resulting in a total of 4 observations for each of these time periods.

#### Total RNA isolation, cDNA synthesis, primer design and ddPCR

Total COC RNA was isolated using the Quick-RNA Microprep Kit (Zymo Research, Irvine, CA, USA) with on-column DNAse treatment per manufacturer. Quantity (Nanodrop ND-1000; NanoDrop Technologies, USA) and quality (RNA Nano LabChip; Bioanalyzer 2100, Agilent, USA) of total RNA were determined (RIN values ranged from 7.2 to 10; median of 8.9). Reverse transcription with oligo (dT) and random primers (500 ng per 20 ul reaction; iScript Reverse Transcription Supermix, Bio-Rad, Hercules, CA, USA) was performed per manufacturer and diluted 5-fold with 1 mM Tris-HCl (pH 8.0) and 0.01 mM EDTA (0.1X TE) before performing digital droplet polymerase chain reactions (ddPCR) analyses. A pool resulting from all samples within each collection day was sham-transcribed (iScript No-RT Control Supermix, Bio-Rad) as an additional control.

Primer-BLAST (National Center for Biotechnology Information; U.S. National Library of Medicine, Bethesda, MD, USA) was utilized to design primers spanning exon-exon junction and/or introns (Table 1). Resulting amplicons were evaluated via gel electrophoresis and sequenced to ensure single product with correct specificity. As per manufacturer guidelines, a gradient of primer concentrations and annealing temperatures were tested to determine optimal conditions (Table 1) that would maximize fluorescent intensity between positive and negative droplets while minimizing occurrence of off-target and/or non-specific amplification events (i.e., rain). Digital droplet PCR was performed in duplicate using 10 ng of nucleic acid per reaction per manufacturer’s instructions. No template controls (NTC; 0.1X TE) were analyzed to assess background signal and control for exogenous contamination. Samples were amplified for 40 cycles, 30s per conditions in Table 1 followed by signal stabilization (4°C for 5 min, 40°C for 5 min, hold at 12°C). Acquired data were analyzed using QuantaSoft Analysis Pro (ver. 1.0, Bio-Rad) to calculate number of copies per µl.

**Table 1:** Primer sequences and annealing conditions used for ddPCR.

**Gene**	**GenBank Accession Number**	**Amplicon Location (bp)**	**Primer Set**	**Primer Concentration (nM)**	**Annealing Temperature (°C)**
*IL6*	NM_173923.2	349-568	3’-GCATCTTCTCCAGCAGGTCAG	250	56
			5’-CAATCTGGGTTCAATCAGGCGAT		
*IL6R*	NM_001110785.3	343-666	3’-TCGGGCTGTAGGAGTTTGTAGC	125	56
			5’-GCGCTTGGTGGTGGATGTTC		
*IL6ST*	XM_010816769.3*	1136-1355	3’-CGCGTCTGATTTGCCAACAA	250	58
			5’-GTCTCATGCTCACGGCACTA		
*LIF*	NM_173931	157-359	3’-CTGGGCCGTGTAATAGAGGAT	250	58
			5’-TCTTGGCGGCAGGAGTTGT		
*SDHA*	NM_174178	1433-1646	3’-TCCGTAGAGGCTGCTGATCT	250	58
			5’-GTCCTGCAGACCCGGAGATA		

* Wooldridge and Ealy (2019).

Transcript abundance was normalized to succinate dehydrogenase A (SDHA). Succinate dehydrogenase A has been used as a normalizer for *in vivo* and *in vitro* matured COCs (Assidi et al., 2010; Macabelli et al., 2014; del Collado et al., 2017; Botigelli et al., 2018) and for heat-stressed COCs (Pavani et al., 2017). It is stably expressed in cumulus during maturation (Assidi et al., 2010; Regassa et al., 2011).

#### Progesterone production

Progesterone released into the maturation medium by COCs matured at 38.5 or 41.0°C (Figure 1) was analyzed by radioimmunoassay per manufacturer’s instructions (Double Antibody RIA; MP Biomedicals, Santa Ana, CA., USA). Assay sensitivity was 0.02 ng/mL; inter- and intra-assay coefficients of variation were 7.6 and 6.0%, respectively.

### Study two: embryo development after COC exposure to 41.0°C for first 4 or 6 hIVM

Because heat-induced increases in *IL6* levels were noted by 4 hIVM and by 4 and 6 hIVM for *IL6ST,* a second study was performed to evaluate consequences of a 41.0 °C exposure for 4 or 6 h on embryonic development. Cumulus-oocyte complexes meeting criteria described above were randomly allocated to three different treatment groups: 38.5 °C for 24 hIVM, 41.0°C for 4 hIVM, or 41.0 °C for 6 hIVM. After 4 or 6 hIVM at 41.0 °C, COCs were transferred to 38.5 °C for remainder of *in vitro* maturation. After a total of 24 hIVM, a combination of frozen-thawed-washed sperm from two bulls was added at ~500,000 motile sperm/ml to each well of COCs. Presumptive zygotes were denuded of cumulus and associated sperm at ~16 to 18 h after addition of sperm. Embryonic cleavage was assessed 66 to 70 h after addition of sperm at which point essential amino acids were added to culture medium. At 172 to 178 h after addition of sperm, blastocyst development was recorded. Blastocyst stage and quality scoring was performed as described by Schrock et al. (2007). Number of nuclei was assessed using fluorescent microscopy (40X magnification using a Nikon Eclipse TE300; UV-2A filter: ex 330 to 380 nm, em 400 to 420 nm; Nikon Instruments, Melville, NY, USA) after fixation in 3% paraformaldehyde. Thereafter, embryos were stained using 5 µg/ml Hoechst 33342, washed, and then mounted on glass slides in Dulbecco’s phosphate buffered saline containing 50% glycerol and 0.5 µg/ml Hoechst 33342. For this study, COCs were collected from ovaries on five different days with total of 1,338 COCs being utilized.

### Statistical analyses

A randomized complete block design was implemented for study one. Data were analyzed using generalized linear mixed models (PROC GLIMMIX, SAS 9.4, SAS Institute, Cary, NC, USA) blocking on day of COC collection. Mindful of the 2 x 9 factorial treatment arrangement, fixed effects in the model included IVM temperature (38.5 and 41.0°C), IVM time (2, 4, 6, 8, 10, 12, 16, 20 and 24 h), and respective interaction (IVM temperature x IVM time; 18 treatment combinations). Treatment differences were determined using Fishers-protected least significant differences and are reported as least squares means ± standard error.

Multisource nonlinear mixed model regression (JMP PRO 14, SAS Institute) was performed *a posteriori* to determine the extent to which maturation of COCs at 41.0°C shifted the timing of changes in transcript abundance compared to levels observed in COCs matured at 38.5°C. The nonlinear prediction model fit was: $a\times e{\;}^{-\left(0.5\;\times {\left(\frac{hIVM-b}{c}\right)}^{2}\right)}$ where a is peak (highest) value, b is when peak value occurred, and c is growth rate (width of temporal production).

Study two implemented a randomized complete block design; mixed model analysis of variance (PROC GLIMMIX; SAS 9.4) was utilized to test the main effect of treatment while blocking on the random effect of day of oocyte collection. Treatment differences were determined using Fishers-protected least significant differences.

## Results

### Study one: Interleukin 6, IL6 receptor, signal transducer and LIF transcripts during *in vitro* maturation in COCs matured at 38.5 or 41.0°C

#### Relative abundance of IL6 in COCs matured at 38.5 and 41.0°C

**Figure 2:**
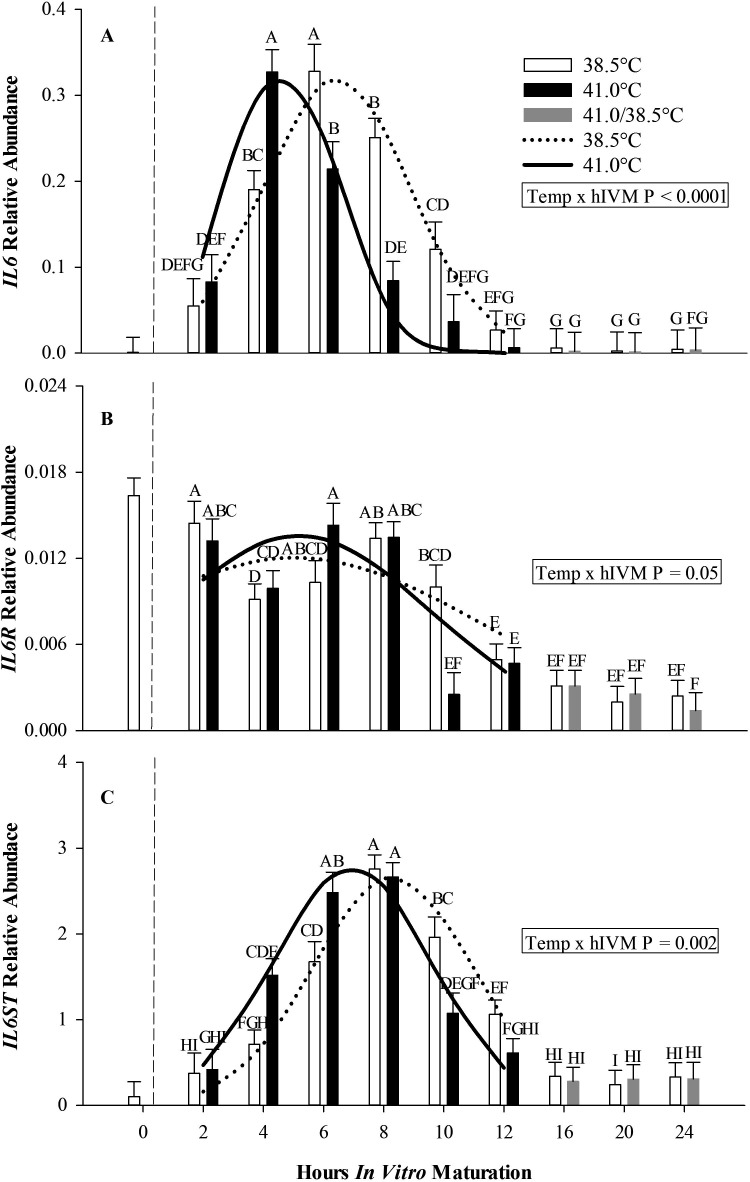
Relative abundance of interleukin 6 and signaling molecules in cumulus-oocyte complexes matured for up to 24 h at either 38.5°C or 41.0°C (first 12 h; 38.5°C thereafter). Interaction of temperature x hIVM between 38.5°C and 41.0°C for *IL6* (Panel A), *IL6R* (Panel B), *IL6ST* (Panel C). Bars (least squares means ± SEM) having different letter designations ^A-I^ differ at indicated *P* value (Temp x hIVM). Dashed (38.5°C) and solid (41.0°C) lines indicate relative abundance curves over the first 12 hIVM.

Abundance of *IL6* transcript differed depending upon IVM temperature and hIVM (Temp x hIVM interaction, P < 0.0001; Figure 2A). While barely detectable soon after COC collection (0 hIVM), *IL6* abundance at 2 hIVM was similar between COCs matured at 38.5 and 41.0°C. By 4 hIVM, acute exposure to 41.0°C increased relative abundance of *IL6* transcript compared to 38.5°C. Interestingly, by 6 and 8 hIVM the relative abundance of *IL6* in COCs exposed to 41.0°C was lower than that observed in COCs matured at 38.5°C. By 12 hIVM, relative abundance of *IL6* was similar between 41.0 and 38.5°C COCs, levels remained low for remainder of maturation.

Use of multisource nonlinear mixed model regression showed that the major consequence of 41.0°C exposure at the beginning of maturation was to shift the temporal production of *IL6*. To this end, *IL6* levels peaked at 4.67 hIVM in COCs directly exposed to 41.0°C, whereas *IL6* levels peaked at 6.44 hIVM when COCs were matured at 38.5°C (P < 0.001; Figure 2A, Table 2). Peak values and growth rate were similar in COCs matured at 38.5 and 41.0°C (Table 2).

**Table 2:** Impact of 41.0°C exposure on IL6, IL6R and IL6ST levels in COCs during *in vitro* maturation using multisource nonlinear mixed model regression.

**Transcript**		**Peak Value** *	**Peak Time (hIVM)**	Growth Rate**
*IL6*	38.5°C	0.32 ± 0.03^a^	6.44 ± 0.23^a^	2.43 ± 0.28^a^
	41.0°C	0.33 ± 0.03^a^	4.67 ± 0.23^b^	1.82 ± 0.22^a^
	P-value	P > 0.05	**P < 0.001**	P > 0.05
	R^2^	0.67		
	SSE	0.41		
*IL6R*	38.5°C	0.01 ± 0.00^a^	5.02 ± 1.49^a^	6.33 ± 2.01^a^
	41.0°C	0.01 ± 0.00^a^	5.16 ± 0.70^a^	4.42 ± 0.82^a^
	P-value	P > 0.05	P > 0.05	P > 0.05
	R^2^	0.33		
	SSE	0.001		
*IL6ST*	38.5°C	2.66 ± 0.19^a^	8.29 ± 0.23^a^	2.66 ± 0.22^a^
	41.0°C	2.77 ± 0.20^a^	6.95 ± 0.23^b^	2.63 ± 0.23^a^
	P-value	P > 0.05	**P < 0.01**	P > 0.05
	R^2^	0.69		
	SSE	22.7		

* Peak value: highest obtained level; **Growth rate: Full Width Half Maximum (3 standard deviations from the mid-point at half maximum). ^a,b^ means differ P < 0.05.

#### Relative abundance of IL6 receptor (IL6R) in COCs matured at 38.5 and 41.0°C

Relative abundance of *IL6R* differed depending on hIVM and IVM temperature (Temp x hIVM interaction, P = 0.05; Figure 2B). Abundance of *IL6R* was highest during the first 8 hIVM with abundance decreasing thereafter and reaching lowest levels by 20 to 24 hIVM. Except for the 10 hIVM time period, *IL6R* levels were similar in COCs matured at 38.5 and 41.0°C (Figure 2B, Table 2).

#### Relative abundance of IL6 Signal Transducer (IL6ST) in COCs matured at 38.5 and 41.0°C

Relative abundance of *IL6ST* transcripts differed depending on IVM temperature and hIVM (Temp x hIVM interaction, P = 0.002; Figure 2C). While barely detectable in COCs soon after collection from antral follicles, *IL6ST* abundance at 2 hIVM was similar between COCs matured at 38.5 and 41.0°C. However, by 4 and 6 hIVM, 41.0°C exposure resulted in higher levels of *IL6ST* compared to 38.5°C counterparts. At 8 hIVM relative abundance of *IL6ST* in COCs matured at 41.0°C was similar to COCs matured at 38.5°C. By 10 hIVM, *IL6ST* was lower in COCs matured at 41.0°C compared to those matured at 38.5 °C. By 16 hIVM, relative abundance of *IL6ST* was similar between 41.0 and 38.5°C and equivalent to levels observed at the onset of maturation (i.e., 2 hIVM).

Use of multisource nonlinear mixed model regression showed that the major consequence of 41.0°C exposure at the beginning of maturation was to shift the temporal production of *IL6ST*. To this end, *IL6ST* peak levels occurred at 6.95 hIVM when COCs were matured at 41.0°C, whereas peak values were noted at 8.29 hIVM when COCs were matured at 38.5°C (P < 0.01; Figure 2C, Table 2). Peak values and growth rates for *IL6ST* were similar in COCs matured at 38.5 and 41.0°C (Table 2).

#### Relative abundance of Leukemia Inhibitory Factor (LIF)

**Figure 3:**
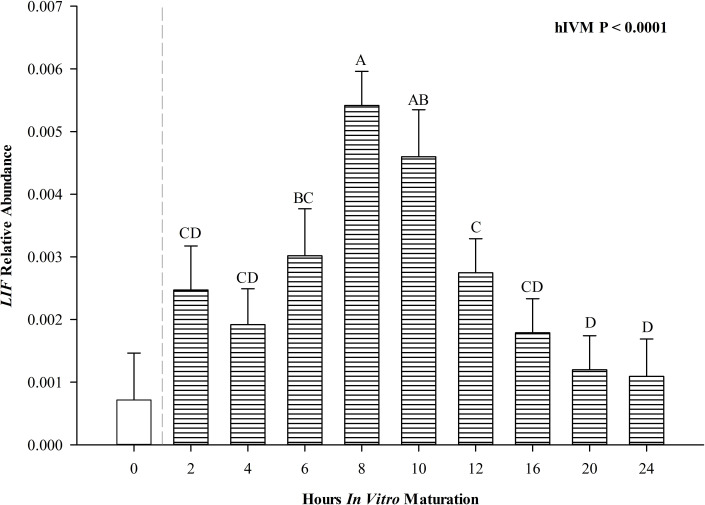
Relative abundance of *LIF* in cumulus-oocyte complexes during maturation, 0 hIVM not included in analysis but as a visual representation of a starting point, averaged across maturation temperatures presented as least squares means ± SEM. ^A-D^ means differ *P* < 0.0001.

Relative abundance of *LIF* transcript changed over time (P < 0.0001, Figure 3) but was not affected by maturation temperature. Soon after collection and placement of COCs in maturation medium, *LIF* levels increased up through 8 hIVM. After 10 hIVM *LIF* levels decreased and by 12 hIVM, relative abundance was similar to values obtained at 2, 4 and 6 hIVM.

#### Progesterone production

**Figure 4:**
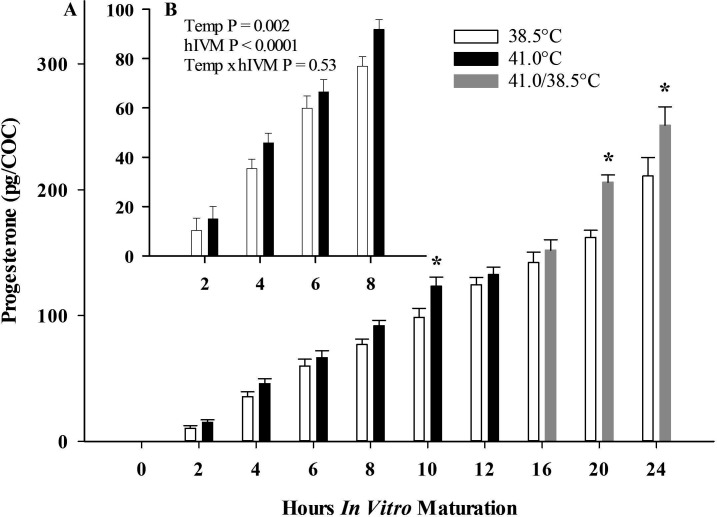
Average progesterone produced per cumulus-oocyte complex (COC) during *in vitro* maturation (IVM) at 38.5°C or 41.0°C as measured in conditioned medium. COCs underwent IVM for up to 24 h at 38.5°C or 41.0°C (first 12 hours; 38.5°C thereafter). (A) Temperature x hIVM P = 0.012; *denotes heat-induced increase in progesterone at indicated time point (B) Impact of 41.0°C on COC on progesterone production during time period when relative abundance of *IL6* was altered by 41.0°C.

Progesterone released per COC into the maturation medium differed depending on IVM temperature and hIVM (Temp x hIVM interaction, P = 0.01; Figure 4A). When all time points were included in the model (2 to 24 hIVM), heat-induced increases in progesterone produced per COC were most prominent at 20 and 24 hIVM (Figure 4A). When including only the time periods when *IL6* levels were shifted by direct exposure to 41.0°C (i.e., 2 to 8 hIVM), progesterone produced per COC was only affected by IVM temperature and hIVM (i.e., no interaction; heat induced differences were not influenced by time; Figure 4B). To this end, progesterone per COC was 45.7 vs 54.9 pg when COCs were matured at 38.5 and 41.0°C, respectively (P = 0.002). Independent of temperature but related to time (hIVM), progesterone per COC was 12.7, 40.7, 63.2 and 84.5 pg at 2, 4, 6 and 8 hIVM, respectively (P < 0.0001).

### Study two: embryo development after COC exposure to 41.0°C for first 4 or 6 hIVM

Ability of COCs to cleave and develop to the blastocyst stage after being exposed to an acute, short-term heat shock of 41.0°C during the first 4 or 6 hIVM was similar to COCs matured at 38.5°C (Table 3). Stage and quality of blastocyst stage embryos from COCs matured at 38.5 or 41.0°C were similar. Number of nuclei in blastocyst stage embryos did not differ when originating from COCs matured at 38.5 °C for 24 h, 41 °C for 4hIVM or 41°C for 6 hIVM (Table 3).

**Table 3:** Impact of an acute exposure to 41.0°C for the first 4 or 6 h of *in vitro* maturation.

**Treatment**	**No. OMM1**	**Cleaved (%)**	**8 to 16-cell (%)**	**Blastocysts (%)**	**Stage**	**Quality**	**Nuclei**
38.5°C-24 h	454	68.87 ± 3.48	74.64 ± 3.47	23.81 ± 2.77	6.69 ± 0.12	1.79 ± 0.12	101.68 ± 11.39
41.0°C-4 h*	444	74.77 ± 3.19	75.19 ± 3.39	29.61 ± 3.08	6.75 ± 0.12	1.96 ± 0.12	118.76 ± 11.39
41.0°C-6 h*	440	75.62 ± 3.07	65.88 ± 3.78	27.73 ± 2.92	6.64 ± 0.12	1.74 ± 0.12	116.07 ± 11.39
P-value		0.1426	0.0714	0.2558	0.6006	0.1586	0.4313

* Exposed to 41.0°C for 4 or 6 h at onset of maturation period followed by 38.5°C for a total of 24 h. ^1^Number of COCs placed in maturation medium (OMM) for indicated treatment

## Discussion

Novel findings described herein provide further insight related to *IL6* and its receptor signaling component transcripts in the bovine cumulus-oocyte complex as it undergoes *in vitro* maturation. Examination at frequent time intervals confirmed temporal expression of *IL6* and *IL6ST* to the first 12 hours. Interestingly, the major impact of an acute, short-term exposure to 41.0°C was to shift the timing of *IL6* and *IL6ST* expression. Specifically, peak *IL6* levels in COCs exposed to an acute heat shock of 41.0°C occurred ~2 hours earlier than thermoneutral COCs. Regarding impact on *IL6ST* expression, peak levels in COCs exposed to 41.0°C occurred ~1.5 hours *earlier* than thermoneutral COCs. Functional significance of these findings remains unclear, but heat-related shifts in *IL6* and *IL6ST* expression may explain heat-induced hastening of meiotic maturation reported previously by our laboratory.

Specific to the COC, both the oocyte and its associated cumulus produce IL6 (bovine: (Tscherner et al., 2018), human: (Zolti et al., 1991; Machelon et al., 1994), murine: (Liu et al., 2009), ovine: (Zhao et al., 2012)). Our study demonstrated that the *IL6* transcript is minimally detectable in germinal vesicle (GV) stage COCs soon after removal from antral follicles. Transcript levels are higher by 2 hIVM and peak at 6.44 hours in bovine COCs matured at 38.5°C. Levels decrease thereafter and remain low for remainder of maturation. Collectively findings of the study described herein document temporal expression of *IL6* in the bovine during the first part of maturation and extend the findings of Tscherner et al. (2018) who examined *IL6* abundance in bovine COCs at only three time periods (0, 7, and 24 hIVM). Consistent with our findings in the bovine, *IL6* was present at low levels in ovine COCs soon after removal from antral follicles (Zhao et al., 2012) with peak expression noted at 4 hIVM; levels decreased thereafter. Use of Western blot confirmed presence of the IL6 protein in ovine COCs soon after removal from the antral follicle with highest levels at 4 and 8 hIVM (Zhao et al., 2012).

Regarding the potential for COC-derived *IL6* to be impactful at the level of the cumulus-oocyte complex, outcomes of study one show that *IL6* receptor abundance is greatest when *IL6* levels are markedly increasing during the first 6 hIVM. Furthermore, receptor levels are lowest during the latter half of maturation (~12 h to 24 hIVM) when *IL6* transcripts remain unchanged and are at low levels. Transcript abundance for the *IL6* receptor relates well to protein levels in ovine COCs (Zhao et al., 2012) which has been localized to the oocyte surface (Zhao et al., 2012). Although receptor binding is important, intracellular signaling related to IL6 is dependent on forming a complex with IL6 signal transducer (Hibi et al., 1990; Mackiewicz et al., 1992; Heinrich et al., 2003; Wolf et al., 2014). Like *IL6*, *IL6ST* was temporally expressed during the first half of maturation, except peak levels occurred ~2 hours later. In murine COCs and hybridoma cells, addition of IL6 increases IL6ST transcript and protein levels (Canellada et al., 2008; Liu et al., 2009). Whether or not COC-derived increases in *IL6* affect *IL6ST* expression in the bovine is unclear. Because of its importance after receptor binding, it is intuitive for *IL6ST* levels to peak and persist a bit longer than *IL6*, which is what we observed in study one.

The presence of this multi-functional cytokine, its receptor, and associated signal transducer set the stage for IL6 to be playing an active and important role within the maturing COC. Although the specific factor(s) underlying the beginnings of maturation are not yet fully elucidated, an increase in COC-derived *IL6* with levels peaking at or around 6 hIVM is likely a significant promotant of GV breakdown (GVBD). In other cell types, IL6 reduces gap junction permeability (Temme et al., 1998) which is requisite for GVBD. When murine COCs are cultured in a hypoxanthine-containing medium to inhibit spontaneous breakdown of the GV, addition of IL6 and its soluble receptor *induced* GVBD (Liu et al., 2009). Marked increases in COC-derived *IL6* peaking at or around 6 hIVM in study one overlap with time period leading up to and when GVBD occurs (Hyttel et al., 1986; Edwards et al., 2005; Hooper et al., 2015; Campen et al., 2018). Interestingly, direct exposure to an elevated temperature of 41.0°C *induces* GVBD in bovine COCs (Edwards et al., 2005; Hooper et al., 2015; Campen et al., 2018) which is consistent with consequences of adding IL6 to meiotically inhibited oocytes (Liu et al., 2009). Heat-induced hastening of GVBD is detectable as early as 4 hIVM and more prominent by 6 hIVM (Hooper et al., 2015). Although factors triggering accelerated GVBD when activated by 41.0°C remain unclear, the heat-induced *shift* in the timing of *IL6* expression and associated IL6ST by ~1.5 to 2 hours (study one) supports the notion for IL6 to be a contributing factor in the heat-induced hastening of GVBD previously reported (Edwards et al., 2005; Hooper et al., 2015; Campen et al., 2018).

Leukemia inhibitory factor, a member of the IL6 family (Nicola and Babon, 2015), promotes oocyte maturation in multiple species (Dang‐Nguyen et al., 2014; Mo et al., 2014; Wang et al., 2019). Unlike *IL6*, there was no impact of 41.0°C on *LIF* expression at any time period examined. Although *LIF* levels increased during the first part of maturation (up through 8 to 10 hIVM) and decreased thereafter, highest levels were reached ~2 hours *after IL6* peaked similar to temporal changes observed herein for *IL6ST*. Both IL6 and LIF depend on IL6ST for receptor-mediated signal transduction, though IL6 complexed with its receptor has higher affinity for IL6ST when both ligands are present (Gearing et al., 1991; Tscherner et al., 2018). Thus, temporal production of *LIF* coinciding with signal transducer expression may be important to influence other developmentally important events for maturation success (e.g., metaphase I and metaphase II progression).

Cumulus-derived progesterone released into the maturation medium increases soon after placement of COCs into medium and continues to increase throughout maturation (Study one, Rispoli et al., 2013; Campen et al., 2018). When examining just the time periods when *IL6* levels were shifted by direct exposure to 41.0°C (i.e., 2 to 8 hIVM), COCs released more progesterone into maturation medium (45.7 vs 54.9 pg for control and heat stress, respectively). Similar findings were previously reported by Campen et al. (2018). Blocking progesterone’s ability to bind to its receptor using RU486 prevented FSH-induction of *IL6* in murine oocytes (Liu et al., 2009) suggesting that progesterone may be a contributory factor helping modulate IL6 production.

Mindful that prolonged exposure (12 or more hours) is detrimental to embryo development (Edwards and Hansen, 1996; Lawrence et al., 2004; Roth and Hansen, 2004a, b; Edwards et al., 2005; Castro and Hansen, 2007; Schrock et al., 2007; Sugiyama et al., 2007; Edwards et al., 2009; Soto and Smith, 2009; Zhandi et al., 2009), an additional study was conducted to examine developmental consequences of 41.0°C when occurring at the beginning of oocyte maturation but for shorter time periods (i.e., first 4 or 6 hIVM). When utilizing COCs collected from antral follicles during the latter part of fall, winter, and through late spring to avoid developmental issues related to summer heat stress (reviewed by Wolfenson and Roth, 2019), blastocyst development was not impaired by 41.0°C. In fact, blastocyst development, stage and quality scores, and nuclei numbers were numerically higher when COCs where acutely exposed to a higher than normal temperature of 38.5°C.

In retrospect, absence of a *negative* effect on embryo development after direct exposure of naïve COCs to an acute-short term heat “shock” is not surprising when occurring at or near the onset of oocyte maturation. Body temperature of females exhibiting estrus is often elevated as a result of heightened levels of sexual activity (Lewis and Newman, 1984; Kyle et al., 1998; Piccione et al., 2003; Fisher et al., 2008; Suthar et al., 2011; Miura et al., 2017; Randi et al., 2018; Higaki et al., 2019). Peak temperature typically occurs at or around the LH surge (Rajamahendran et al., 1989; Mosher et al., 1990; Fisher et al., 2008) which is important to induce ovulation and maturation of the oocyte resident within the ovulatory follicle.

## Conclusion

In summary, heat-induced shift in the temporal production of *IL6* along with its impact on progesterone likely cooperate in heat-induced hastening of meiotic progression described by others. Given potency of an acute exposure to directly alter components important to promote meiotic maturation, it is not surprising that elevated body temperature occurring at inappropriate and for extended time periods during chronic periods of summer heat stress or disease reduce pregnancy outcomes by directly affecting cumulus-oocyte components.

## References

[B001] Armstrong DV (1994). Heat stress interaction with shade and cooling. J Dairy Sci.

[B002] Assidi M, Dieleman SJ, Sirard MA (2010). Cumulus cell gene expression following the LH surge in bovine preovulatory follicles: potential early markers of oocyte competence. Reproduction.

[B003] Botigelli RC, Razza EM, Pioltine EM, Fontes PK, Schwarz KRL, Leal CLV, Nogueira MFG (2018). Supplementing *in vitro* embryo production media by NPPC and sildenafil affect the cytoplasmic lipid content and gene expression of bovine cumulus-oocyte complexes and embryos. Reprod Biol.

[B004] Campen KA, Abbott CR, Rispoli LA, Payton RR, Saxton AM, Edwards JL (2018). Heat stress impairs gap junction communication and cumulus function of bovine oocytes. J Reprod Dev.

[B005] Canellada A, Alvarez I, Berod L, Gentile T (2008). Estrogen and progesterone regulate the IL-6 signal transduction pathway in antibody secreting cells. J Steroid Biochem Mol Biol.

[B006] Castro EPLA, Hansen PJ (2007). Interactions between oxygen tension and glucose concentration that modulate actions of heat shock on bovine oocytes during *in vitro* maturation. Theriogenology.

[B007] Clark A, Matos DG, Jackson JA, Palmer SS, Tran CAT (2011). Use of IL-6 type cytokines for maturation of oocytes. Appl. No. 10/998,080:Patent No. 8,071,375 B072.

[B008] Dang‐Nguyen TQ, Haraguchi S, Kikuchi K, Somfai T, Bodó S, Nagai T (2014). Leukemia inhibitory factor promotes porcine oocyte maturation and is accompanied by activation of signal transducer and activator of transcription 3. Mol Reprod Dev.

[B009] del Collado M, Silveira JC, Sangalli JR, Andrade GM, Sousa LRDS, Silva LA, Meirelles FV, Perecin F (2017). Fatty acid binding protein 3 and transzonal projections are involved in lipid accumulation during *in vitro* maturation of bovine oocytes. Sci Rep.

[B010] Dunlap S, Vincent C (1971). Influence of postbreeding thermal stress on conception rate in beef cattle. J Anim Sci.

[B011] Ealy AD, Drost M, Hansen PJ (1993). Developmental changes in embryonic resistance to adverse effects of maternal heat stress in cows. J Dairy Sci.

[B012] Edwards JL, Bogart AN, Rispoli LA, Saxton AM, Schrick FN (2009). Developmental competence of bovine embryos from heat-stressed ova. J Dairy Sci.

[B013] Edwards JL, Hansen PJ (1996). Elevated temperature increases heat shock protein 70 synthesis in bovine two-cell embryos and compromises function of maturing oocytes. Biol Reprod.

[B014] Edwards JL, Saxton AM, Lawrence JL, Payton RR, Dunlap JR (2005). Exposure to a physiologically relevant elevated temperature hastens *in vitro* maturation in bovine oocytes. J Dairy Sci.

[B015] Elvinger F, Hansen P, Natzke R (1991). Modulation of function of bovine polymorphonuclear leukocytes and lymphocytes by high temperature *in vitro* and *in vivo.*. Am J Vet Res.

[B016] Fallon G (1962). Body temperature and fertilization in the cow. J Reprod Fertil.

[B017] Faundez R, Kawecka O, Aniołek O, Petrajtis-Gołobów M, Karska D, Gajewski Z (2015). The influence of GM-CSF, IL-6 and TGF-α on *in vitro* maturation of porcine oocytes. Reprod Domest Anim.

[B018] Faundez R, Kawecka O, Karska D, Petrajtis M, Gajewski Z (2014). The influence of GM-CSF, IL-6 and TGF-α on the *in vitro* maturation of bovine oocytes. Reprod Domest Anim.

[B019] Fisher AD, Morton R, Dempsey JM, Henshall JM, Hill JR (2008). Evaluation of a new approach for the estimation of the time of the LH surge in dairy cows using vaginal temperature and electrodeless conductivity measurements. Theriogenology.

[B020] Gaalaas R (1945). Effect of atmospheric temperature on body temperature and respiration rate of Jersey cattle. J Dairy Sci.

[B021] Gearing D, Thut C, VandeBos T, Gimpel S, Delaney P, King J, Price V, Cosman D, Beckmann M (1991). Leukemia inhibitory factor receptor is structurally related to the IL‐6 signal transducer, gp130. EMBO J.

[B022] Heinrich PC, Behrmann I, Haan S, Hermanns HM, Muller-Newen G, Schaper F (2003). Principles of interleukin (IL)-6-type cytokine signalling and its regulation. Biochem J.

[B023] Hibi M, Murakami M, Saito M, Hirano T, Taga T, Kishimoto T (1990). Molecular cloning and expression of an IL-6 signal transducer, gp130. Cell.

[B024] Higaki S, Miura R, Suda T, Andersson LM, Okada H, Zhang Y, Itoh T, Miwakeichi F, Yoshioka K (2019). Estrous detection by continuous measurements of vaginal temperature and conductivity with supervised machine learning in cattle. Theriogenology.

[B025] Hooper LM, Payton RR, Rispoli LA, Saxton AM, Edwards JL (2015). Impact of heat stress on germinal vesicle breakdown and lipolytic changes during *in vitro* maturation of bovine oocytes. J Reprod Dev.

[B026] Hyttel P, Xu K, Smith S, Greve T (1986). Ultrastructure of *in-vitro* oocyte maturation in cattle. J Reprod Fertil.

[B027] Kyle BL, Kennedy AD, Small JA (1998). Measurement of vaginal temperature by radiotelemetry for the prediction of estrus in beef cows. Theriogenology.

[B028] Lawrence JL, Payton RR, Godkin JD, Saxton AM, Schrick FN, Edwards JL (2004). Retinol improves development of bovine oocytes compromised by heat stress during maturation. J Dairy Sci.

[B029] Lewis GS, Newman SK (1984). Changes throughout estrous cycles of variables that might indicate estrus in dairy cows. J Dairy Sci.

[B030] Liu Z, Matos DG, Fan H-Y, Shimada M, Palmer S, Richards JS (2009). Interleukin-6: an autocrine regulator of the mouse cumulus cell-oocyte complex expansion process. Endocrinology.

[B031] Macabelli CH, Ferreira RM, Gimenes LU, de Carvalho NAT, Soares JG, Ayres H, Ferraz ML, Watanabe YF, Watanabe OY, Sangalli JR, Smith LC, Baruselli PS, Meirelles FV, Chiaratti MR (2014). Reference gene selection for gene expression analysis of oocytes collected from dairy cattle and buffaloes during winter and summer. PLoS One.

[B032] Machelon V, Emilie D, Lefevre A, Nome F, Durand-Gasselin I, Testart J (1994). Interleukin-6 biosynthesis in human preovulatory follicles: some of its potential roles at ovulation. J Clin Endocrinol Metab.

[B033] Mackiewicz A, Schooltink H, Heinrich PC, Rose-John S (1992). Complex of soluble human IL-6-receptor/IL-6 up-regulates expression of acute-phase proteins. J Immunol.

[B034] Miura R, Yoshioka K, Miyamoto T, Nogami H, Okada H, Itoh T (2017). Estrous detection by monitoring ventral tail base surface temperature using a wearable wireless sensor in cattle. Anim Reprod Sci.

[B035] Mo X, Wu G, Yuan D, Jia B, Liu C, Zhu S, Hou Y (2014). Leukemia inhibitory factor enhances bovine oocyte maturation and early embryo development. Mol Reprod Dev.

[B036] Mosher M, Ottobre J, Haibel G, Zartman D (1990). Estrual rise in body temperature in the bovine II. The temporal relationship with ovulation. Anim Reprod Sci.

[B037] Nicola NA, Babon JJ (2015). Leukemia inhibitory factor (LIF). Cytokine Growth Factor Rev.

[B038] Pavani KC, Rocha A, Baron E, Lourenço J, Faheem M, Silva FM (2017). The effect of kinetic heat shock on bovine oocyte maturation and subsequent gene expression of targeted genes. Zygote.

[B039] Piccione G, Caola G, Refinetti R (2003). Daily and estrous rhythmicity of body temperature in domestic cattle. BMC Physiol.

[B040] Rajamahendran R, Robinson J, Desbottes S, Walton JS (1989). Temporal relationships among estrus, body temperature, milk yield, progesterone and luteinizing hormone levels, and ovulation in dairy cows. Theriogenology.

[B041] Randi F, McDonald M, Duffy P, Kelly AK, Lonergan P (2018). The relationship between external auditory canal temperature and onset of estrus and ovulation in beef heifers. Theriogenology.

[B042] Regassa A, Rings F, Hoelker M, Cinar U, Tholen E, Looft C, Schellander K, Tesfaye D (2011). Transcriptome dynamics and molecular cross-talk between bovine oocyte and its companion cumulus cells. BMC Genomics.

[B043] Rispoli LA, Edwards JL, Pohler KG, Russell S, Somiari RI, Payton RR, Schrick FN (2019). Heat-induced hyperthermia impacts the follicular fluid proteome of the periovulatory follicle in lactating dairy cows. PLoS One.

[B044] Rispoli LA, Lawrence JL, Payton RR, Saxton AM, Schrock GE, Schrick FN, Middlebrooks BW, Dunlap JR, Parrish JJ, Edwards JL (2011). Disparate consequences of heat stress exposure during meiotic maturation: embryo development after chemical activation vs fertilization of bovine oocytes. Reproduction.

[B045] Rispoli LA, Payton RR, Gondro C, Saxton AM, Nagle KA, Jenkins BW, Schrick FN, Edwards JL (2013). Heat stress effects on the cumulus cells surrounding the bovine oocyte during maturation: altered matrix metallopeptidase 9 and progesterone production. Reproduction.

[B046] Roman-Ponce H, Thatcher W, Buffington D, Wilcox C, Van Horn H (1977). Physiological and production responses of dairy cattle to a shade structure in a subtropical environment. J Dairy Sci.

[B047] Roth Z, Hansen PJ (2004). Involvement of apoptosis in disruption of developmental competence of bovine oocytes by heat shock during maturation. Biol Reprod.

[B048] Roth Z, Hansen PJ (2004). Sphingosine 1-phosphate protects bovine oocytes from heat shock during maturation. Biol Reprod.

[B049] Schrock GE, Saxton AM, Schrick FN, Edwards JL (2007). Early *in vitro* fertilization improves development of bovine ova heat stressed during *in vitro* maturation. J Dairy Sci.

[B050] Seath D, Miller G (1946). The relative importance of high temperature and high humidity as factors influencing respiration rate, body temperature, and pulse rate of dairy cows. J Dairy Sci.

[B051] Soto P, Smith LC (2009). BH4 peptide derived from Bcl-xL and Bax-inhibitor peptide suppresses apoptotic mitochondrial changes in heat stressed bovine oocytes. Mol Reprod Dev.

[B052] Sugiyama S, McGowan M, Phillips N, Kafi M, Young M (2007). Effects of increased ambient temperature during IVM and/or IVF on the *in vitro* development of bovine zygotes. Reprod Domest Anim.

[B053] Suthar VS, Burfeind O, Patel JS, Dhami AJ, Heuwieser W (2011). Body temperature around induced estrus in dairy cows. J Dairy Sci.

[B054] Temme A, Traub O, Willecke K (1998). Downregulation of connexin32 protein and gap-junctional intercellular communication by cytokine-mediated acute-phase response in immortalized mouse hepatocytes. Cell Tissue Res.

[B055] Tscherner A, Brown AC, Stalker L, Kao J, Dufort I, Sirard M-A, LaMarre J (2018). STAT3 signaling stimulates miR-21 expression in bovine cumulus cells during *in vitro* oocyte maturation. Sci Rep.

[B056] Turner H (1982). Genetic variation of rectal temperature in cows and its relationship to fertility. Anim Sci.

[B057] Ulberg LC, Burfening PJ (1967). Embryo death resulting from adverse environment on spermatozoa or ova. J Anim Sci.

[B058] Wang J, Liu Z, Sun Q, Xia S, Cui J, Yang L, An L, Zhang J, Su L, Su Y, Du F (2019). Combined treatment with cysteamine and leukemia inhibitory factor promotes guinea pig oocyte meiosis *in vitro.*. Am J Transl Res.

[B059] Wang Y, Liang N, Yao G, Tian H, Zhai Y, Yin Y, Sun F (2014). Knockdown of TrkA in cumulus oocyte complexes (COCs) inhibits EGF-induced cumulus expansion by down-regulation of IL-6. Mol Cell Endocrinol.

[B060] Wolf J, Rose-John S, Garbers C (2014). Interleukin-6 and its receptors: a highly regulated and dynamic system. Cytokine.

[B061] Wolfenson D, Roth Z (2019). Impact of heat stress on cow reproduction and fertility. Anim Front.

[B062] Wooldridge LK, Ealy AD (2019). Interleukin-6 increases inner cell mass numbers in bovine embryos. BMC Dev Biol.

[B063] Zakari A, Molokwu E, Osori D (1981). Effects of rectal and ambient temperatures and humidity on conception rates. Theriogenology.

[B064] Zhandi M, Towhidi A, Nasr-Esfahani MH, Eftekhari-Yazdi P, Zare-Shahneh A (2009). Unexpected detrimental effect of insulin like growth factor-1 on bovine oocyte developmental competence under heat stress. J Assist Reprod Genet.

[B065] Zhao X, Cang M, Gao X-y, Yang M, Yuan J, Zhu B, Wang Z, Liu D (2012). Expression of interleukin–6 and interleukin-6 receptor in ovine oocytes during *in vitro* maturation. J Integr Agric.

[B066] Zolti M, Ben-Rafael Z, Meirom R, Shemesh M, Bider D, Mashiach S, Apte RN (1991). Cytokine involvement in oocytes and early embryos. Fertil Steril.

